# Supplementary material for: Unmasking Racial, Ethnic, and Socioeconomic Disparities in United States Chordoma Clinical Trials: Systematic Review
Source: Cancers (Basel). 2025 Jan 12;17(2):225. doi: 10.3390/cancers17020225 (PMC11763698; doi:10.3390/cancers17020225)
Supplement: Supplementary file 1 [file cancers-17-00225-s001.zip › Supplementary materials - US Chordoma clinical trials disparity.pdf]

Supplementary materials

Prospective Study Protocol

- **P**atients
  - Patient suffering from Chordoma
- **I**ntervention
  - Radiotherapy
  - Systemic therapy
- **C**omparator
  - None
- **O**utcome
  - Racial data[1]
    - White / Caucasians
    - Black / African American
    - Asians
    - American Indian or Alaska Native
    - Native Hawaiian or Other Pacific Islander
    - More than one race
    - Unknown or Not Reported
  - Ethnic data[1]
    - Hispanic or Latino
    - Not Hispanic or Latino
    - Unknown or Not Reported
  - Socioeconomic indicators
    - Insurance status
    - Income
    - Employment status
    - Occupation
    - Primary language
    - Social Vulnerability Index
    - Area Deprivation Index
- **S**tudy design
  - Inclusion:
    - Randomized controlled clinical trials
    - Non-randomized clinical trials
  - Exclusion:
    - Preclinical studies
    - Cohort studies

[1] Defined according to the Food and Drug Administration reporting guidelines

        - Case-control studies
        - Case series
        - Case reports
        - Letter to editor, commentaries and all other such types of correspondences
        - Literature reviews

MeSH terms were acquired for aforementioned relevant terms from the MeSH resource of NCBI including "Chordoma"[MeSH Terms].[1]

Search strategy implemented on: 19th of August, 2024

- **PubMed**
    - ("Chordoma"[MeSH Terms]) AND (clinicaltrial[Filter] OR randomizedcontrolledtrial[Filter]) **54**
- **CSDR**
    - MeSH descriptor: [Chordoma] explode all trees **26**
- **Epistemonikos**
    - (title:(Chordoma) OR abstract:(Chordoma)) **8**
- **ClinicalTrials**
    - Completed chordoma trials with results **7**

- Total hits: 95
- Article(s) identified from manual exploration: 1 **[Bavarian Nordic. A Phase 2 Trial of BN-Brachyury and Radiation Therapy in Patients With Advanced Chordoma [Internet]. clinicaltrials.gov; 2023 Mar [cited 2024 Jan 1]. Report No.: NCT03595228. Available from: https://clinicaltrials.gov/study/NCT03595228]**
    - Duplicates removed: 23
        - Total documents screened: 73
            - **Trials included: 5**

Documents excluded:

- **Trial protocol with no results published (n = 8)**
    - Peking University People's Hospital. A Prospective, Multicentre, Open-Label, Randomised Phase 2 Trial to Study the Efficacy and Safety of Anlotinib Hydrochloride Versus Imatinib Mesylate in Locally Advanced, Unresectable or Metastatic Chordoma. clinicaltrials.gov; 2019. Accessed November 8, 2024. https://clinicaltrials.gov/study/NCT04042597

- Uhl M, Edler L, Jensen AD, et al. Randomized phase II trial of hypofractionated proton versus carbon ion radiation therapy in patients with sacrococcygeal chordoma-the ISAC trial protocol. Radiat Oncol Lond Engl. 2014;9:100. doi:10.1186/1748-717X-9-100
- Nikoghosyan AV, Karapanagiotou-Schenkel I, Münter MW, Jensen AD, Combs SE, Debus J. Randomised trial of proton vs. carbon ion radiation therapy in patients with chordoma of the skull base, clinical phase III study HIT-1-Study. BMC Cancer. 2010;10:607. doi:10.1186/1471-2407-10-607
- Centre Antoine Lacassagne. Analysis of the Toxicity and Efficacy of Daily 1 vs 2 Beam Proton Therapy : Analysis of the Toxicity and Efficacy of Daily 1 vs 2 Beam Proton Therapy. clinicaltrials.gov; 2024. Accessed November 8, 2024. https://clinicaltrials.gov/study/NCT06029218
- ImmunityBio, Inc. QUILT-3.091 NANT Chordoma Vaccine: A Randomized Phase 1b/2 Trial of the NANT Chordoma Vaccine vs. Radiation in Subjects With Unresectable Chordoma. clinicaltrials.gov; 2021. Accessed November 8, 2024. https://clinicaltrials.gov/study/NCT03647423
- M.D. Anderson Cancer Center. Phase II Evaluation of Proton Beam Therapy for Skull Base Chordoma. clinicaltrials.gov; 2024. Accessed November 8, 2024. https://clinicaltrials.gov/study/NCT00496119
- Italian Sarcoma Group. Title of Study: SAcral Chordoma: A Randomized & Observational Study on Surgery Versus Definitive Radiation Therapy in Primary Localized Disease (SACRO). clinicaltrials.gov; 2023. Accessed November 8, 2024. https://clinicaltrials.gov/study/NCT02986516
- Gillies MJ, Lyon PC, Wu F, et al. High-intensity focused ultrasonic ablation of sacral chordoma is feasible: a series of four cases and details of a national clinical trial. Br J Neurosurg. 2017;31(4):446-451. doi:10.1080/02688697.2016.1267330

- **Different study design (n = 17)**
  - Akmansu M, Kurt G, Demircan V, Senturk E. Results of Chordoma Patients Treated by Different Approaches in a Single Institution. Turk Neurosurg. 2020;30(3):366-370. doi:10.5137/1019-5149.JTN.24406-19.4
  - Champagne PO, Passeri T, Jabre R, Bernat AL, Voormolen EH, Froelich S. Vertebrobasilar Artery Encasement by Skull Base Chordomas: Surgical Outcome and Management Strategies. Oper Neurosurg Hagerstown Md. 2020;19(4):375-383. doi:10.1093/ons/opaa091
  - Goitein M, Cox JD. Should randomized clinical trials be required for proton radiotherapy? J Clin Oncol Off J Am Soc Clin Oncol. 2008;26(2):175-176. doi:10.1200/JCO.2007.14.4329
  - Radaelli S, Fossati P, Stacchiotti S, et al. The sacral chordoma margin. Eur J Surg Oncol J Eur Soc Surg Oncol Br Assoc Surg Oncol. 2020;46(8):1415-1422. doi:10.1016/j.ejso.2020.04.028
  - Oh HC, Hong CK, Lee KS, et al. Apparent diffusion coefficient as a prognostic factor in clival chordoma. Sci Rep. 2021;11(1):486. doi:10.1038/s41598-020-79894-8

- Zhou H, Jiang L, Wei F, et al. Prognostic Factors in Surgical Patients with Chordomas of the Cervical Spine: A Study of 52 Cases from a Single Institution. Ann Surg Oncol. 2017;24(8):2355-2362. doi:10.1245/s10434-017-5884-5
- Hobusch GM, Bodner F, Walzer S, et al. C-reactive protein as a prognostic factor in patients with chordoma of lumbar spine and sacrum--a single center pilot study. World J Surg Oncol. 2016;14:111. doi:10.1186/s12957-016-0875-8
- Cheney MD, Chen YL, Lim R, et al. [18F]-Fluoromisonidazole positron emission tomography/computed tomography visualization of tumor hypoxia in patients with chordoma of the mobile and sacrococcygeal spine. Int J Radiat Oncol Biol Phys. 2014;90(5):1030-1036. doi:10.1016/j.ijrobp.2014.08.016
- Chibbaro S, Cornelius JF, Froelich S, et al. Endoscopic endonasal approach in the management of skull base chordomas--clinical experience on a large series, technique, outcome, and pitfalls. Neurosurg Rev. 2014;37(2):217-224; discussion 224-225. doi:10.1007/s10143-013-0503-9
- Taniguchi M, Kohmura E. Endoscopic endonasal removal of laterally extended clival chordoma using side-viewing scopes. Acta Neurochir (Wien). 2012;154(4):627-632. doi:10.1007/s00701-011-1225-9
- Guan J yue, Wang B bei, Wei C jun, Li Y hao. [Percutaneous intratumoral injection with pingyangmycin lipiodol emulsion for treatment of recurrent sacrococcygeal chordomas]. Nan Fang Yi Ke Da Xue Xue Bao. 2010;30(10):2366-2369.
- Choi D, Melcher R, Harms J, Crockard A. Outcome of 132 operations in 97 patients with chordomas of the craniocervical junction and upper cervical spine. Neurosurgery. 2010;66(1):59-65; discussion 65. doi:10.1227/01.NEU.0000362000.35742.3D
- Liu AL, Wang ZC, Sun SB, Wang MH, Luo B, Liu P. Gamma knife radiosurgery for residual skull base chordomas. Neurol Res. 2008;30(6):557-561. doi:10.1179/174313208X297878
- Noël G, Feuvret L, Dhermain F, et al. [Chordomas of the base of the skull and upper cervical spine. 100 patients irradiated by a 3D conformal technique combining photon and proton beams]. Cancer Radiother J Soc Francaise Radiother Oncol. 2005;9(3):161-174. doi:10.1016/j.canrad.2005.04.005
- Imai R, Kamada T, Tsuji H, et al. Carbon ion radiotherapy for unresectable sacral chordomas. Clin Cancer Res Off J Am Assoc Cancer Res. 2004;10(17):5741-5746. doi:10.1158/1078-0432.CCR-04-0301
- Kockro RA, Serra L, Tseng-Tsai Y, et al. Planning and simulation of neurosurgery in a virtual reality environment. Neurosurgery. 2000;46(1):118-135; discussion 135-137.
- Waisman M, Kligman M, Roffman M. Posterior approach for radical excision of sacral chordoma. Int Orthop. 1997;21(3):181-184. doi:10.1007/s002640050146

- **Different patient population (n = 33)**

- Mai J. Phase IV Trial Evaluating the Use of Stereotactic Body Radiotherapy for the Treatment of Spine Metastases and Primary Spine Tumors. clinicaltrials.gov; 2020. Accessed January 1, 2024. https://clinicaltrials.gov/study/NCT01347307
- Intensity Therapeutics, Inc. A Phase 1/2 Safety Study of Intratumorally Administered INT230-6 in Adult Subjects With Advanced Refractory Cancers. clinicaltrials.gov; 2024. Accessed November 7, 2024. https://clinicaltrials.gov/study/NCT03058289
- Sarcoma Alliance for Research through Collaboration. A Phase II Trial of Dasatinib in Advanced Sarcomas. clinicaltrials.gov; 2018. Accessed November 7, 2024. https://clinicaltrials.gov/study/NCT00464620
- Frazier R. Phase IV Trial to Use Stereotactic Body Radiotherapy for Head and Neck Tumors. clinicaltrials.gov; 2020. Accessed January 1, 2024. https://clinicaltrials.gov/study/NCT01344356
- BioMed Valley Discoveries, Inc. Phase I Safety Study of Intratumoral Injection of Clostridium Novyi-NT Spores in Patients With Treatment-Refractory Solid Tumor Malignancies. clinicaltrials.gov; 2019. Accessed November 7, 2024. https://clinicaltrials.gov/study/NCT01924689
- Suit HD, Goitein M, Munzenrider J, et al. Increased efficacy of radiation therapy by use of proton beam. Strahlenther Onkol Organ Dtsch Rontgengesellschaft Al. 1990;166(1):40-44.
- Chernichenko VA, Tolstopiatov BA, Konovalenko VF, Monich AI, Palivets AI. [The combination treatment of malignant bone tumors using fast neutrons]. Vopr Onkol. 1990;36(8):970-973.
- Austin-Seymour M, Urie M, Munzenrider J, et al. Considerations in fractionated proton radiation therapy: clinical potential and results. Radiother Oncol J Eur Soc Ther Radiol Oncol. 1990;17(1):29-35. doi:10.1016/0167-8140(90)90046-y
- Marucci L, Niemierko A, Liebsch NJ, Aboubaker F, Liu MCC, Munzenrider JE. Spinal cord tolerance to high-dose fractionated 3D conformal proton-photon irradiation as evaluated by equivalent uniform dose and dose volume histogram analysis. Int J Radiat Oncol Biol Phys. 2004;59(2):551-555. doi:10.1016/j.ijrobp.2003.10.058
- Santoni R, Liebsch N, Finkelstein DM, et al. Temporal lobe (TL) damage following surgery and high-dose photon and proton irradiation in 96 patients affected by chordomas and chondrosarcomas of the base of the skull. Int J Radiat Oncol Biol Phys. 1998;41(1):59-68. doi:10.1016/s0360-3016(98)00031-5
- Baumann BC, Lustig RA, Mazzoni S, et al. A prospective clinical trial of proton therapy for chordoma and chondrosarcoma: Feasibility assessment. J Surg Oncol. 2019;120(2):200-205. doi:10.1002/jso.25502
- Liebsch NJ. Randomized Study of Charged Particle RT for Chordomas and Chondrosarcomas of the Base of Skull or Cervical Spine. clinicaltrials.gov; 2017. Accessed November 8, 2024. https://clinicaltrials.gov/study/NCT00592748

- Schuetze SM, Bolejack V, Choy E, et al. Phase 2 study of dasatinib in patients with alveolar soft part sarcoma, chondrosarcoma, chordoma, epithelioid sarcoma, or solitary fibrous tumor. Cancer. 2017;123(1):90-97. doi:10.1002/cncr.30379
- Bilaniuk LT, Zimmerman RA, Wehrli FW, et al. Cerebral magnetic resonance: comparison of high and low field strength imaging. Radiology. 1984;153(2):409-414. doi:10.1148/radiology.153.2.6541355
- Hospices Civils de Lyon. Transnational Randomized Study Comparing Carbon Ions Therapy Versus Conventional Radiotherapy - Including Protontherapy - for the Treatment of Radioresistant Tumors. clinicaltrials.gov; 2021. Accessed November 8, 2024. https://clinicaltrials.gov/study/NCT02838602
- Welzel T, Meyerhof E, Uhl M, et al. Diagnostic accuracy of DW MR imaging in the differentiation of chordomas and chondrosarcomas of the skull base: A 3.0-T MRI study of 105 cases. Eur J Radiol. 2018;105:119-124. doi:10.1016/j.ejrad.2018.05.026
- Heery CR, Singh BH, Rauckhorst M, et al. Phase I Trial of a Yeast-Based Therapeutic Cancer Vaccine (GI-6301) Targeting the Transcription Factor Brachyury. Cancer Immunol Res. 2015;3(11):1248-1256. doi:10.1158/2326-6066.CIR-15-0119
- DeLaney TF, Liebsch NJ, Pedlow FX, et al. Long-term results of Phase II study of high dose photon/proton radiotherapy in the management of spine chordomas, chondrosarcomas, and other sarcomas. J Surg Oncol. 2014;110(2):115-122. doi:10.1002/jso.23617
- Uhl M, Welzel T, Oelmann J, et al. Active raster scanning with carbon ions: reirradiation in patients with recurrent skull base chordomas and chondrosarcomas. Strahlenther Onkol Organ Dtsch Rontgengesellschaft Al. 2014;190(7):686-691. doi:10.1007/s00066-014-0608-2
- Adenis A, Ray-Coquard I, Italiano A, et al. A dose-escalating phase I of imatinib mesylate with fixed dose of metronomic cyclophosphamide in targeted solid tumours. Br J Cancer. 2013;109(10):2574-2578. doi:10.1038/bjc.2013.648
- Tuan J, Vischioni B, Fossati P, et al. Initial clinical experience with scanned proton beams at the Italian National Center for Hadrontherapy (CNAO). J Radiat Res (Tokyo). 2013;54 Suppl 1(Suppl 1):i31-42. doi:10.1093/jrr/rrt036
- Haddad H, Dejean C, Henriques de Figueiredo B, et al. [Helical tomotherapy for axial and paraspinal tumours: experience of Institut Bergonié (14 cases)]. Cancer Radiother J Soc Francaise Radiother Oncol. 2011;15(5):404-412. doi:10.1016/j.canrad.2011.03.005
- Schlampp I, Karger CP, Jäkel O, et al. Temporal lobe reactions after radiotherapy with carbon ions: incidence and estimation of the relative biological effectiveness by the local effect model. Int J Radiat Oncol Biol Phys. 2011;80(3):815-823. doi:10.1016/j.ijrobp.2010.03.001
- Schulz-Ertner D, Nikoghosyan A, Didinger B, Debus J. Carbon ion radiation therapy for chordomas and low grade chondrosarcomas--current status of the

clinical trials at GSI. Radiother Oncol J Eur Soc Ther Radiol Oncol. 2004;73 Suppl 2:S53-56. doi:10.1016/s0167-8140(04)80014-8

- Chugh R, Dunn R, Zalupski MM, et al. Phase II study of 9-nitro-camptothecin in patients with advanced chordoma or soft tissue sarcoma. J Clin Oncol Off J Am Soc Clin Oncol. 2005;23(15):3597-3604. doi:10.1200/JCO.2005.02.170
- Schulz-Ertner D, Nikoghosyan A, Thilmann C, et al. Results of carbon ion radiotherapy in 152 patients. Int J Radiat Oncol Biol Phys. 2004;58(2):631-640. doi:10.1016/j.ijrobp.2003.09.041
- Schulz-Ertner D, Nikoghosyan A, Thilmann C, et al. Carbon ion radiotherapy for chordomas and low-grade chondrosarcomas of the skull base. Results in 67 patients. Strahlenther Onkol Organ Dtsch Rontgengesellschaft Al. 2003;179(9):598-605. doi:10.1007/s00066-003-1120-2
- Schulz-Ertner D, Haberer T, Jäkel O, et al. Radiotherapy for chordomas and low-grade chondrosarcomas of the skull base with carbon ions. Int J Radiat Oncol Biol Phys. 2002;53(1):36-42. doi:10.1016/s0360-3016(01)02827-9
- Debus J, Haberer T, Schulz-Ertner D, et al. [Carbon ion irradiation of skull base tumors at GSI. First clinical results and future perspectives]. Strahlenther Onkol Organ Dtsch Rontgengesellschaft Al. 2000;176(5):211-216. doi:10.1007/s000660050002
- Jho HD, Carrau RL. Endoscopic endonasal transsphenoidal surgery: experience with 50 patients. J Neurosurg. 1997;87(1):44-51. doi:10.3171/jns.1997.87.1.0044
- Kumon Y, Zenke K, Ohta S, Hatakeyama T, Sakaki S, Yanagihara N. [Operative results in fourteen cases of paranasal sinus and anterior cranial fossa lesions surgically treated by an extended transbasal approach]. No Shinkei Geka. 1995;23(10):889-895.
- Zhou L, Guo H, Li S, Ji Y, Huang F. An extensive subfrontal approach to the lesions involving the skull base. Chin Med J (Engl). 1995;108(6):407-412.
- Wilson CB, Boldrey EB, Enot KJ. 1,3-bis (2-chloroethyl)-1-nitrosourea (NSC-409962) in the treatment of brain tumors. Cancer Chemother Rep. 1970;54(4):273-281.

- **Undertaken in countries other than USA (n = 10)**

  - Seidensaal K, Froehlke A, Lentz-Hommertgen A, et al. Hypofractionated proton and carbon ion beam radiotherapy for sacrococcygeal chordoma (ISAC): An open label, randomized, stratified, phase II trial. Radiother Oncol J Eur Soc Ther Radiol Oncol. 2024;198:110418. doi:10.1016/j.radonc.2024.110418
  - Le Cesne A, Chevreau C, Perrin C, et al. Regorafenib in patients with relapsed advanced or metastatic chordoma: results of a non-comparative, randomised, double-blind, placebo-controlled, multicentre phase II study. ESMO Open. 2023;8(3):101569. doi:10.1016/j.esmoop.2023.101569
  - Stacchiotti S, Morosi C, Lo Vullo S, et al. Imatinib and everolimus in patients with progressing advanced chordoma: A phase 2 clinical study. Cancer. 2018;124(20):4056-4063. doi:10.1002/cncr.31685

- Lebellec L, Bertucci F, Tresch-Bruneel E, et al. Circulating vascular endothelial growth factor (VEGF) as predictive factor of progression-free survival in patients with advanced chordoma receiving sorafenib: an analysis from a phase II trial of the french sarcoma group (GSF/GETO). Oncotarget. 2016;7(45):73984-73994. doi:10.18632/oncotarget.12172
- Bompas E, Le Cesne A, Tresch-Bruneel E, et al. Sorafenib in patients with locally advanced and metastatic chordomas: a phase II trial of the French Sarcoma Group (GSF/GETO). Ann Oncol Off J Eur Soc Med Oncol. 2015;26(10):2168-2173. doi:10.1093/annonc/mdv300
- Stacchiotti S, Tamborini E, Lo Vullo S, et al. Phase II study on lapatinib in advanced EGFR-positive chordoma. Ann Oncol Off J Eur Soc Med Oncol. 2013;24(7):1931-1936. doi:10.1093/annonc/mdt117
- Mammar H, Kerrou K, Nataf V, et al. Positron emission tomography/computed tomography imaging of residual skull base chordoma before radiotherapy using fluoromisonidazole and fluorodeoxyglucose: potential consequences for dose painting. Int J Radiat Oncol Biol Phys. 2012;84(3):681-687. doi:10.1016/j.ijrobp.2011.12.047
- Stacchiotti S, Longhi A, Ferraresi V, et al. Phase II study of imatinib in advanced chordoma. J Clin Oncol Off J Am Soc Clin Oncol. 2012;30(9):914-920. doi:10.1200/JCO.2011.35.3656
- Imai R, Kamada T, Tsuji H, et al. Effect of carbon ion radiotherapy for sacral chordoma: results of Phase I-II and Phase II clinical trials. Int J Radiat Oncol Biol Phys. 2010;77(5):1470-1476. doi:10.1016/j.ijrobp.2009.06.048
- Stacchiotti S, Marrari A, Tamborini E, et al. Response to imatinib plus sirolimus in advanced chordoma. Ann Oncol Off J Eur Soc Med Oncol. 2009;20(11):1886-1894. doi:10.1093/annonc/mdp210
